# Effect of Dental CAD-CAM Resin Composite Thickness on the Polymerization Behavior of Dual-Cure Resin Cements for Endocrown Restoration

**DOI:** 10.3390/ma19061217

**Published:** 2026-03-19

**Authors:** Yuya Komagata, Takafumi Watanabe, Shinji Yoshii, Chihiro Masaki, Hiroshi Ikeda

**Affiliations:** 1 Division of Biomaterials, Kyushu Dental University, Kitakyushu 803-8580, Japan; pty_pty_kjm@yahoo.co.jp (Y.K.); r16ikeda@fa.kyu-dent.ac.jp (H.I.); 2 Division of Occlusion and Maxillofacial Reconstruction, Kyushu Dental University, Kitakyushu 803-8580, Japan; 3 Division of Promoting Learning Design Education, Kyushu Dental University, Kitakyushu 803-8580, Japan; r08yoshii@fa.kyu-dent.ac.jp; 4 Division of Oral Reconstruction and Rehabilitation, Kyushu Dental University, Kitakyushu 803-8580, Japan; masaki@kyu-dent.ac.jp

**Keywords:** CAD-CAM material, dual-cure resin cement, light-cure resin cement, degree of conversion, hardness, endocrown

## Abstract

**Highlights:**

**What are the main findings?**

- Light transmission through CAD-CAM resin composites decreases exponentially with thickness.
- Dual-cure resin cement polymerization decreases markedly above 5.5 mm thickness.
- Adequate polymerization occurs when the composite thickness is below approximately 5.5 mm.

**What are the implications of the main findings?**

- Self-curing alone cannot compensate for insufficient light under thick restorations.
- Excessive pulp-chamber extension may increase the risk of debonding and failure.

**Abstract:**

This study investigated the effect of CAD-CAM resin composite thickness on the polymerization behavior of dual-cure resin cements used for endocrown restorations. Three commercially available dual-cure resin cements and one light-cure resin cement (for comparison) were polymerized by light irradiation through CAD-CAM resin composite plates of varying thicknesses (1.5, 3.5, 5.5, 7.5, and 9.5 mm). Transmitted light intensity was measured using an optical spectrometer. Polymerization behavior was evaluated immediately after irradiation and after 24 h of aging using Fourier transform infrared spectroscopy to determine the degree of conversion (DC) and Vickers hardness (VH) testing. Transmitted light intensity decreased logarithmically with increasing composite thickness, with less than 1% of incident light reaching the resin cement at thicknesses ≥ 5.5 mm. For the dual-cure resin cements, DC and VH values significantly decreased when the composite thickness exceeded 5.5 mm. Although DC and VH increased after 24 h due to self-curing, values beneath thicker composites remained lower than those beneath 1.5 mm thick composites. The light-cure resin cement failed to polymerize when the composite thickness exceeded 7.5 mm. These results indicate that CAD-CAM resin composite thickness critically influences resin cement polymerization, highlighting the importance of thickness control in endocrown restorations.

## 1. Introduction

An endocrown is a type of full-coverage dental restoration designed for endodontically treated teeth [1]. Unlike conventional crowns, endocrowns exhibit a thicker structure and a monolithic design that integrates both the crown and core, with the core portion extending into the pulp chamber [2]. This configuration provides reliable mechanical strength and retention without the need for a separate post-and-core buildup, thereby enabling minimal tooth reduction and improved preservation of sound tooth structure. Consequently, the selection of appropriate restorative materials and adhesive systems is a critical determinant for achieving optimal mechanical performance and long-term clinical durability of endocrown restorations [3,4].

Endocrown prostheses are typically fabricated using computer-aided design and computer-aided manufacturing (CAD-CAM) technology [5,6,7] with prefabricated block materials. Among these CAD-CAM materials, ceramic-based materials have been widely used for endocrowns because of their excellent mechanical strength, physicochemical stability, and biocompatibility [8]. Glass ceramics, including feldspathic porcelain, leucite-reinforced glass, and lithium disilicate glass, have been extensively employed for endocrown fabrication [9,10,11,12]. In particular, lithium disilicate glass has achieved widespread clinical use owing to its high strength and durable bonding with resin-based luting agents (resin cements), resulting in favorable long-term clinical outcomes [13,14,15]. More recently, zirconia-reinforced lithium disilicate has attracted increasing attention as an alternative material due to its enhanced mechanical properties [16,17]. Zirconia ceramics have also been applied to endocrowns because of their superior strength and toughness, which contribute to long-term stability in the oral environment [18,19]. However, despite their excellent mechanical properties, ceramic materials—including both glass ceramics and zirconia—exhibit mechanical incompatibility with natural tooth structures, particularly in terms of elastic modulus and hardness. These discrepancies can lead to stress concentration within the abutment tooth and excessive wear of the opposing dentition [20,21,22,23]. Such mechanical incompatibility remains one of the major limitations of zirconia- and glass-ceramic-based endocrowns. In contrast, CAD-CAM resin composites have recently gained attention as alternative materials for endocrowns because of their superior mechanical compatibility with natural tooth tissues compared with ceramics [24,25,26]. The hardness of these composites is lower than that of enamel, thereby reducing the risk of wear on the opposing dentition [27,28,29]. Furthermore, their elastic modulus is close to that of dentin, allowing for more favorable stress distribution and improved mechanical integration with the abutment tooth [30,31]. These characteristics suggest that CAD-CAM resin composites may be advantageous restorative materials for endocrowns in clinical applications [32,33].

Adhesive bonding is another critical factor for the clinical success of endocrown restorations [34]. Dual-cure resin cements are commonly used to bond endocrowns to abutment teeth because they polymerize via two mechanisms: light-curing, which is initiated by light irradiation, and self-curing (chemical curing), which gradually progresses over time. In conventional crown restorations, light-curing is first performed, followed by self-curing to ensure complete polymerization. Because conventional crowns typically have a thickness of approximately 1.5 mm, sufficient light can penetrate the restorative material to initiate polymerization of the underlying resin cement [35]. In contrast, endocrowns often exhibit greater thicknesses (≥3.5 mm), resulting in substantial light scattering and attenuation as light passes through the restorative material [36]. Consequently, only a limited amount of light reaches the resin cement layer, leading to incomplete light polymerization. This insufficient polymerization may compromise bond strength and increase the risk of early debonding or fracture of both the restoration and the abutment tooth. To address this issue, several studies have attempted to optimize bonding protocols and curing strategies for dual-cure resin cements, alongside fundamental investigations into their polymerization behavior [34,36,37,38]. Previous studies have demonstrated that increasing the thickness of restorative materials used for endocrowns—such as lithium disilicate, feldspathic porcelain, and polymer-infiltrated ceramics—significantly reduces the polymerization efficiency of the underlying resin cements [38]. However, limited information is available regarding the influence of CAD-CAM resin composite thickness on the polymerization behavior of dual-cure resin cements.

The present study aimed to clarify the effect of CAD-CAM resin composite thickness on the polymerization behavior of dual-cure resin cements. The degree of conversion (DC) and Vickers hardness (VH) were evaluated as indicators of polymerization efficiency. The null hypothesis of this study was that the thickness of the CAD-CAM resin composite does not affect the DC or VH of the dual-cure resin cements.

## 2. Materials and Methods

### 2.1. Materials

Table 1 lists the three commercial dual-cure resin-based luting agents (resin cements) used in this study. For comparison, one light-cure resin cement was also examined.

Cerasmart 300 (A3 color high-translucent grade, GC Corp., Tokyo, Japan) was used for the CAD-CAM resin composite block. The as-received block was sectioned into plates of different thicknesses (1.5, 3.5, 5.5, 7.5, and 9.5 mm) using a diamond wheel saw under continuous water cooling. The surfaces of the plates were sequentially polished with silicon-carbide abrasive papers (#400, #800, and #1500). Each plate thickness was measured using a digital vernier caliper (CD-15CPX, Mitutoyo Corp., Kawasaki, Japan), and the deviation from the target thickness was maintained within ±0.1 mm. The prepared plates were cleaned ultrasonically in distilled water, then air-dried with an oil-free air blower. These plates of various thicknesses were subsequently used for the transmitted light intensity measurements and polymerization experiments described below.

### 2.2. Transmitted Light Measurement

**Figure 1:**
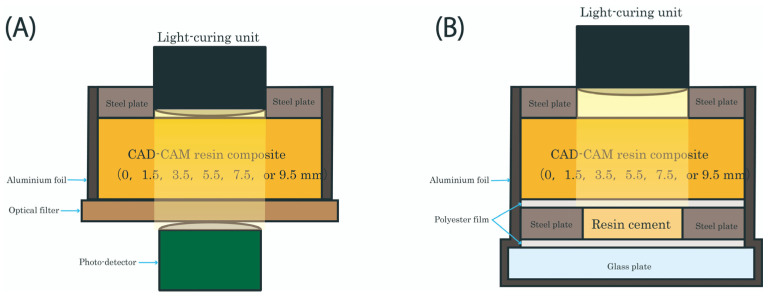
(**A**) Schematic illustration of the experimental setup for measuring the light intensity transmitted through CAD-CAM resin composite plates of different thicknesses. (**B**) Schematic illustration of the experimental setup for polymerization of the resin cement under light irradiation through the CAD-CAM resin composite plate of different thicknesses using a dental light-curing unit.

Figure 1A shows the experimental setup used to measure the light intensity transmitted through CAD-CAM resin composite plates of different thicknesses. A stainless-steel spacer with a circular aperture (10 mm in diameter) was used to standardize the irradiation area and ensure reproducible alignment between the curing light and the composite specimen. The light guide tip (12 mm in diameter) was placed in direct contact with the spacer surface and aligned perpendicular to the specimen surface to minimize angular deviation and variability in irradiance distribution. The light was irradiated through the perforations using a handheld dental light-curing unit (VALO GRAND, Ultradent Products Inc., South Jordan, UT, USA). The light-curing unit was operated in high-power mode, and the irradiance (1600 mW/cm^2^) was verified using a calibrated dental radiometer (Bluephase Meter II, Ivoclar, Schaan, Liechtenstein) at the specimen plane prior to experimentation. The light guide tip diameter was 12 mm, and the tip was positioned in direct contact with the composite plate during irradiation. The emission wavelength range of the curing unit (approximately 395–480 nm) overlaps with the absorption spectra of commonly used photoinitiators in dental resin cements, thereby ensuring spectral compatibility with the materials tested in this study. The light transmitted through the composite resin was detected by an optical spectrometer (FLAME-S-XR1-ES, Ocean Optics Inc., Dunedin, FL, USA) equipped with a neutral density attenuating filter (AND-40C-001, Sigmakoki Co., Ltd., Tokyo, Japan). The attenuating filter was used to prevent detector saturation because the incident light intensity exceeded the upper detection limit of the spectrometer. The maximum peak value of each recorded spectrum was defined as the transmitted peak spectral irradiance. This value was normalized to the corresponding peak value obtained without an interposed composite plate, which was set at 100%, and the transmitted value for each specimen was expressed as a relative percentage. Seven samples were measured for each group (*n* = 7).

### 2.3. Polymerization Test

The light-polymerization test was conducted following the procedure described in our previous study [38]. A schematic illustration of the experimental setup is shown in Figure 1B. The resin cement was placed into a 10 mm-diameter circular cavity within a stainless-steel spacer (1 mm thickness) to ensure a uniform cement layer thickness. A polyester film was placed over the resin cement, and a CAD-CAM resin composite plate of varying thickness (1.5, 3.5, 5.5, 7.5, or 9.5 mm) was mounted on top of the film. The assembly was covered with aluminum foil to prevent exposure to ambient light, leaving only the upper surface open for light irradiation. The handheld dental light-curing unit was placed in contact with the upper surface of the plate, and continuous light irradiation was performed for 90 s at an intensity of 1600 mW/cm^2^. For the direct-light (0 mm) group, light irradiation was applied directly to the resin cement without an interposed composite plate. For the no-light (dark cure) group, no light irradiation was applied, and specimens were maintained under light-shielded conditions. After irradiation, the cured resin cements were immediately evaluated and designated as the “Immediate group.” Independent sets of specimens were stored in a dark incubator at 37 °C for 24 h after irradiation and designated as the “Aged group.” The polymerization behavior of the resin cement was subsequently evaluated using Fourier transform infrared spectroscopy (FTIR) and Vickers hardness testing, as described in the following sections.

### 2.4. Fourier Transform Infrared (FTIR) Spectroscopy

The polymerization behavior of the resin cement under each experimental condition was evaluated by determining the degree of conversion (DC) using Fourier transform infrared (FTIR) spectroscopy. Measurements were performed with an attenuated total reflectance (ATR) FTIR spectrometer (IRSpirit equipped with QATR-S unit; Shimadzu Corp., Tokyo, Japan). To calculate the DC, the absorbance intensities of two characteristic bands—approximately 1638 cm^−1^ (C=C stretching vibration) and 1720 cm^−1^ (C=O stretching vibration)—were obtained before and after polymerization. ATR-FTIR measurements were performed over a spectral range of 650–4000 cm^−1^ with a resolution of 4 cm^−1^ and 50 scans per spectrum. Each spectrum was recorded at its defined time point (either immediately after irradiation or after 24 h of dark storage). To ensure reproducible contact pressure and positioning, the specimen surface was placed in direct contact with the diamond ATR crystal and compressed using the dedicated pressure clamp integrated into the ATR accessory. All spectra were baseline-corrected prior to analysis, and absorbance values were obtained using standardized peak-height measurements within a consistent spectral window. The degree of conversion (DC) was calculated from the ratio of the absorbance peak of the aliphatic C=C bond at approximately 1638 cm^−1^ to that of the carbonyl (C=O) stretching band at approximately 1720 cm^−1^ used as an internal reference. The C=O band was selected because it is consistently present in methacrylate-based resin matrices and remains chemically stable during polymerization. Peak deconvolution or curve-fitting procedures were not applied because the selected absorption bands were clearly distinguishable in the obtained spectra without significant overlap.

The DC was then calculated using the following equation [39,40]:

$$Degree\;of\;conversion\;(\%)=\left(1-\frac{{\left[A(C=C)/A(C=O)\right]}_{after\;polymerization}}{{\left[A(C=C)/A(C=O)\right]}_{before\;polymerization}}\right)\times 100$$

where A(C=C) and A(C=O) represent the absorbance intensities of the C=C and C=O bonds, respectively. Seven samples were measured for each group (*n* = 7).

### 2.5. Vickers Hardness (VH) Measurement

After FTIR analysis, the same specimens were subjected to Vickers hardness testing without additional surface treatment, such as polishing or grinding, in order to preserve the original polymerized surface condition. Vickers hardness measurements were performed on the upper surface of the cement layer, which was in direct contact with the CAD-CAM resin composite specimen (restoration side). This surface corresponds to the clinically relevant region where polymerization is primarily influenced by light transmitted through the restorative material. Measurements were performed using a microhardness tester (HMV-G21ST, Shimadzu Corp., Kyoto, Japan) under a load of 200 g with a dwell time of 15 s. For each experimental group, seven specimens were tested to determine the VH value. Seven samples were measured for each group (*n* = 7).

### 2.6. Statistical Analysis

Statistical analyses were performed using R version 4.5.2 (R Foundation for Statistical Computing, Vienna, Austria). Normality and homogeneity of variance were assessed using the Shapiro–Wilk test and Levene’s test, respectively. In several experimental groups, particularly at greater composite thicknesses, all seven specimens (*n* = 7) remained completely uncured and were unmeasurable. These conditions were designated as “U.C.” (uncured, *n* = 0 for measurable endpoints), resulting in structurally missing data; therefore, a balanced factorial design could not be achieved. In addition, normality was rejected in some groups with a relatively small sample size (*n* = 7). Therefore, non-parametric statistical methods were applied. Differences among composite thickness groups were analyzed using the Kruskal–Wallis test followed by Dunn’s post hoc test with Holm adjustment for multiple comparisons. Differences between the immediate and aged groups were evaluated using the Wilcoxon rank-sum test for independent samples. Statistical significance was set at *p* < 0.05.

## 3. Results

**Figure 2:**
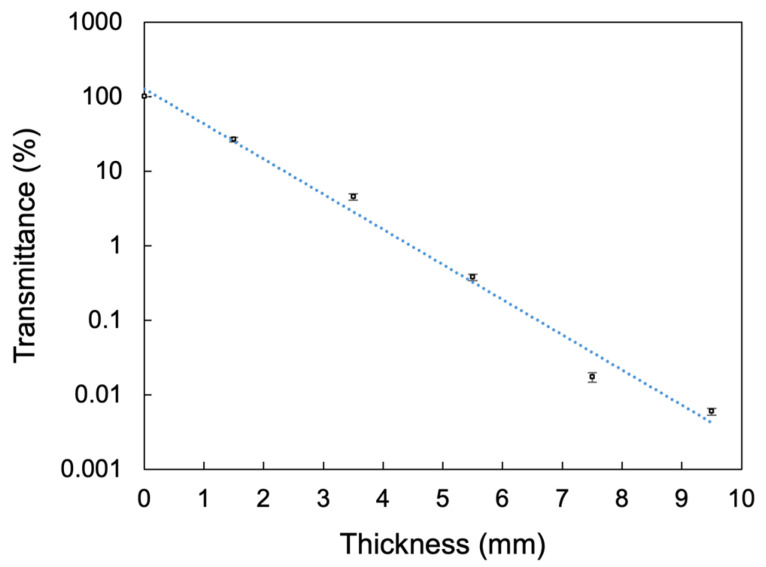
Light intensity transmitted through CAD-CAM resin composite plates of various thicknesses.

Figure 2 shows the light intensity transmitted through CAD-CAM resin composite plates of various thicknesses. The transmitted light intensity decreased logarithmically with increasing composite thickness. This indicates that the amount of light reaching the resin cement decreases markedly as the composite becomes thicker. In particular, at thicknesses of 5.5 mm or greater, light attenuation was substantial, with less than 1% of the irradiated light reaching the resin cement layer.

**Figure 3:**
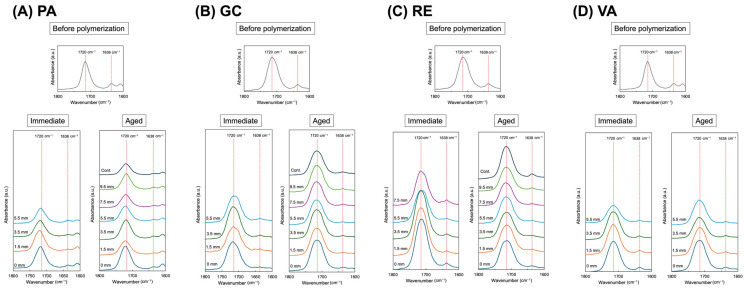
Typical FTIR spectra of the resin cements before polymerization, immediately after light polymerization, and after light-polymerization followed by 24 h of aging. Each luting agent is shown as follows: (**A**) PA, (**B**) GC, (**C**) RE, and (**D**) VA. Missing spectra indicate specimens that could not be measured because the resin cement remained uncured.

Figure 3 shows typical FTIR spectra of the resin cements before polymerization, immediately after light polymerization, and after light polymerization followed by 24 h of aging. Table 2 and Table 3 summarize the degree of conversion (DC) and Vickers hardness (VH) values, respectively, of the resin cements polymerized beneath composites of different thicknesses. For the dual-cure resin cements (PA, GC, and RE), similar polymerization behaviors were observed. Immediately after light irradiation, the DC and VH values of the 1.5 mm thick groups were statistically comparable to those of the direct-light group (0 mm, without composite). However, both DC and VH values decreased progressively as composite thickness increased. A statistically significant reduction was observed at thicknesses of 5.5 mm or greater compared with the direct-light control group. Some specimens polymerized under thicker composites did not harden sufficiently, preventing DC and VH measurements. After 24 h of aging, both DC and VH values increased in specimens polymerized beneath composites 5.5 mm thick or greater, indicating that additional self-curing reactions occurred over time. Nevertheless, the DC and VH values in these groups remained lower than those of the 1.5 mm group, suggesting that self-curing alone was insufficient to achieve complete polymerization. Collectively, these findings indicate that the maximum composite thickness ensuring adequate polymerization of the dual-cure resin cements is approximately 3.5–5.5 mm, whereas polymerization becomes inadequate beyond 7.5 mm.

For the light-cure resin cement (VA), no significant differences were observed in either DC or VH among specimens polymerized under composites with thicknesses ranging from 0 to 5.5 mm. However, when the composite thickness exceeded 7.5 mm, polymerization did not occur. Because this material lacks self-curing capability, no further increase in DC or VH was observed after aging.

## 4. Discussion

The present study investigated the polymerization behaviors of three dual-cure resin cements light-cured through CAD-CAM resin composites of various thicknesses (1.5–9.5 mm) and subsequently self-cured after 24 h of aging, using the degree of conversion (DC) and Vickers hardness (VH) as indicators. The results demonstrated that both DC and VH values of the resin cements immediately after light irradiation decreased as the composite thickness increased. In particular, specimens polymerized through composites thicker than 5.5 mm exhibited a statistically significant reduction in DC and VH. After 24 h of aging, both DC and VH values increased in all groups because of continued self-curing reactions; however, the values for thicker specimens remained significantly lower than those for thinner specimens (1.5 mm). Therefore, the null hypothesis that composite thickness would not affect the polymerization of dual-cure resin cements was rejected.

CAD-CAM resin composites have recently attracted increasing attention for use in endocrown restorations. Several clinical studies have demonstrated the clinical feasibility of endocrowns fabricated from CAD-CAM resin composites [41,42,43,44]. For instance, Vervack et al. [42] investigated molar and premolar endocrowns fabricated from CAD-CAM resin composites and reported a five-year survival rate of 87.5%. Keskin et al. [44] reported a three-year survival rate of 82.7% for premolar and molar restorations. Although these outcomes are clinically acceptable, they remain lower than those reported for lithium disilicate glass endocrowns [8,45]. To further improve the clinical performance of CAD-CAM resin composite endocrowns, fundamental investigations focusing on mechanical compatibility, durability, and the bonding performance of resin cements are required. Several in vitro mechanical studies have shown that the fracture resistance of CAD-CAM resin composite endocrowns is comparable to that of lithium disilicate glass endocrowns, despite the lower intrinsic strength of the composite material [46]. These findings suggest that CAD-CAM resin composites possess adequate mechanical performance for endocrown applications. Regarding bonding behavior, previous studies have examined various bonding protocols for composite endocrowns, including cement thickness, cement space, and structural design [4,47,48,49]. However, the appropriate thickness of CAD-CAM resin composite endocrowns necessary to achieve sufficient curing of the resin cement has not been clearly established. The present study demonstrated that dual-cure resin cements can be effectively polymerized through CAD-CAM resin composites when the material thickness is below approximately 5.5 mm. These findings indicate that the thickness of the CAD-CAM resin composite endocrown is a crucial factor influencing the bonding efficacy of the dual-cure resin cement. The decrease in polymerization observed under thicker CAD-CAM resin composites can be attributed to light attenuation within the composite, caused by absorption and scattering of light within the resin matrix and at the resin–filler interfaces [50]. These findings are consistent with previous studies reporting that increased restorative material thickness significantly reduces light transmission and compromises the polymerization efficiency of underlying resin-based luting agents, particularly in thick indirect restorations [51,52]. This phenomenon follows Lambert–Beer’s law, which states that light intensity decreases exponentially with increasing material thickness. Consequently, when the composite is thick, the transmitted light intensity becomes insufficient to initiate complete polymerization of the underlying resin cement. Although dual-cure resin cements are designed to polymerize through both light- and self-curing mechanisms, the results of this study indicate that self-curing alone did not fully compensate for insufficient light-curing. Previous studies have also reported that the combination of light- and self-curing is significantly more effective than self-curing alone in achieving complete polymerization [53,54,55]. Therefore, to ensure clinical reliability and bonding durability, dual-cure resin cements should be light-cured as thoroughly as possible.

Our previous study [38] investigated the influence of CAD-CAM restorative material type (lithium disilicate, resin composite, and polymer-infiltrated ceramic), thickness, and translucency on the polymerization behavior of a single dual-cure resin cement, focusing primarily on restorative-material-dependent optical behavior. In contrast, the present study focused specifically on CAD-CAM resin composites and examined how thickness-dependent light attenuation affects the polymerization behavior of multiple resin cement systems. This approach allows evaluation of whether the observed attenuation effect occurs consistently across different resin cement formulations. Furthermore, the present study focused specifically on CAD-CAM resin composites, which have recently attracted increasing attention as restorative materials for endocrowns due to their favorable mechanical compatibility with natural tooth structures. Therefore, the objective of this study was to clarify the thickness-dependent polymerization behavior of resin cements under resin composite-based restorations rather than to compare different classes of CAD-CAM materials. The present study used Cerasmart 300 HT (A3 shade) as a representative CAD-CAM resin composite. The spectral emission range of the light-curing unit overlaps with the absorption ranges of common photoinitiators; however, part of the emitted light may be absorbed or scattered within the composite due to the presence of fillers and pigments. Consequently, the transmitted irradiance decreases with increasing composite thickness. Because CAD-CAM resin composites differ in filler systems, translucency grades, and pigment composition, wavelength-dependent light attenuation may vary among brands. Therefore, the material selection of CAD-CAM resin composites may influence the polymerization behavior of resin cements cured through these restorations.

A resin cement thickness of 1.0 mm used in this experiment was intentionally selected as a standardized experimental model. This thickness allowed reliable specimen handling and accurate evaluation by FTIR spectroscopy and Vickers hardness testing. Clinically, the cement film thickness for adhesive luting procedures is generally reported to be approximately 50–150 μm, which is substantially thinner than the thickness used in this study. However, in the present experimental setup, measurements were performed on the upper surface of the cement layer directly contacting the CAD-CAM resin composite specimen. This region is clinically relevant because it represents the interface where light transmission through the restorative material primarily governs polymerization. Therefore, although the absolute thickness exceeded typical clinical cement films, the evaluation focused on the surface most influenced by transmitted light, minimizing the impact of bulk thickness on the measured polymerization parameters.

The three dual-cure resin cements used in this study represent commonly used clinical systems with different compositions, including variations in monomer formulations, filler content, and dual-curing initiator systems. These compositional differences may influence polymerization kinetics and the balance between photo- and self-curing reactions. However, despite these variations, the present results demonstrated a consistent reduction in degree of conversion and hardness with increasing composite thickness. This finding suggests that light attenuation through the restorative material plays a dominant role in determining polymerization efficiency. Resin cements contain photoinitiators that play a critical role in determining polymerization efficiency under wavelength-dependent light attenuation. However, the detailed photoinitiator compositions of the resin cements used in this study are not fully disclosed by the manufacturers. In general, camphorquinone, which is commonly used in dental resin cements, exhibits an absorption maximum in the blue-light region (approximately 460–470 nm), whereas alternative photoinitiators such as trimethylbenzoyl-diphenylphosphine oxide (TPO) predominantly absorb in the near-violet region (approximately 380–420 nm). Because shorter wavelengths are more susceptible to scattering and absorption within resin-based restorative materials, near-violet light components may be attenuated more strongly as the thickness of the restorative material increases. Therefore, when selecting resin cements for endocrown restorations, consideration of the photoinitiator system may be clinically relevant, particularly in cases involving increased restoration thickness. The resin cements polymerized beneath thicker composites (≥5.5 mm) demonstrated significantly lower DC and VH values than those polymerized under thinner composites (1.5 mm). Clinically, a 1.5 mm thickness corresponds to a conventional crown, whereas 5.5 mm represents a typical endocrown configuration. Based on these findings, the present study suggests that the dual-cure resin cement used for endocrowns may not achieve the same level of polymerization or hardness as that used for conventional crowns. Insufficiently cured cements are more susceptible to water sorption, hydrolytic degradation, and mechanical fatigue in the oral environment, which can ultimately lead to loss of adhesion, debonding, and fracture of the endocrown restoration. Therefore, from a clinical perspective, the thickness of CAD-CAM resin composite endocrowns should be carefully designed to ensure optimal polymerization of dual-cure resin cements. A typical endocrown has an overall thickness consisting of about 1.5 mm of coronal structure and 2.0 mm of pulp-chamber extension. As demonstrated in this study, adequate polymerization of the resin cement may not be achieved when the total thickness exceeds approximately 5.5 mm. In particular, the pulp-chamber extension should not be excessively deep, as an unnecessarily thick endocrown can hinder light transmission, compromise cement polymerization, and consequently promote microleakage and the development of secondary caries.

In cases where endocrown thickness exceeds approximately 5.5 mm, alternative curing strategies, such as chemically cured resin cements [56] or touch-cure systems [57,58], may warrant consideration based on previous studies. However, further experimental investigation is required to validate their effectiveness under such conditions. The present study has certain limitations related to the specimen geometry, which was simplified to represent the endocrown structure. In this investigation, a flat composite plate model was intentionally selected to isolate the effect of restorative material thickness under controlled and reproducible conditions. However, this geometry does not reproduce the complex three-dimensional anatomy of clinical endocrowns, where material thickness varies spatially, and light incidence occurs at multiple angles. In actual endocrowns, cusp morphology and pulpal chamber extension may create localized regions with greater thickness and reduced light access. Furthermore, oblique light incidence and internal scattering may lead to non-uniform irradiance distribution. As a result, the present experimental design may overestimate the uniformity of light transmission and polymerization efficiency compared with clinical conditions. Therefore, the identified “critical thickness” range should be interpreted within the limitations of this simplified model. Future studies incorporating anatomically realistic three-dimensional endocrown geometries and multi-directional light irradiation are necessary to more accurately simulate clinical scenarios. In addition, the present findings provide only a fundamental evaluation of polymerization behavior and do not include a direct assessment of bonding performance. Further studies incorporating bond strength measurements and interfacial durability testing are necessary to fully assess the clinical performance of resin-cemented endocrown restorations.

## 5. Conclusions

Within the limitations of the present study, which evaluated specific CAD-CAM resin composite materials, dual-cure resin cements, and defined light-curing conditions using a standardized transmitted-light geometry, the following conclusions can be drawn:

- Light transmission through the tested CAD-CAM resin composites decreased exponentially with increasing material thickness, resulting in substantial attenuation at thicknesses ≥ 5.5 mm under the present irradiation conditions.
- The polymerization efficiency of the evaluated dual-cure resin cements, assessed by degree of conversion and Vickers hardness at the restoration–cement interface, was significantly reduced beneath thicker composite layers.
- Although chemical (self) curing contributed to additional post-irradiation polymerization, it did not fully compensate for reduced light exposure under increased composite thickness in the present experimental model.
- Under the tested materials and curing settings, the experimental threshold thickness for maintaining adequate polymerization was observed between approximately 3.5 and 5.5 mm, whereas polymerization parameters were markedly reduced beyond 7.5 mm.

## Figures and Tables

**Table 1:** Dual-cure resin cements (PA, GC, and RE) and light-cure resin cement (VA) used in this study. The material compositions are based on information provided by the manufacturers.

Code	Cement Type	Product Name	Manufacturer
PA	Dual-curing	Panavia V5	Kuraray Noritake Dental, Tokyo, Japan
GC	Dual-curing	G-CEM LinkForce	GC, Tokyo, Japan
RE	Dual-curing	RelyX Universal Resin	3M, Saint Paul, MN, USA
VA	Light-curing	Variolink Esthetic LC	Ivoclar Vivadent, Schaan, Liechtenstein

**Table 2:** Degree of conversion (DC, %) of dual-cure resin cements (PA, GC, RE) and light-cure resin cement (VA) polymerized by light irradiation through CAD-CAM resin composite plates of different thicknesses in the immediate and aged groups, as determined by FTIR analysis. The direct-light (0 mm) group represents specimens directly irradiated without an interposed CAD-CAM resin composite plate. The no-light (dark cure) group represents specimens polymerized without light irradiation. Values represent means ± standard deviations. Values followed by different uppercase and lowercase letters indicate statistically significant differences among plate thicknesses within the immediate and aged groups, respectively (Dunn’s post hoc test with Holm adjustment, *p* < 0.05). Groups sharing the same letter do not differ significantly. An asterisk (*) indicates statistically significant differences between the immediate and aged groups at the same plate thickness and for the same luting agent (Wilcoxon rank-sum test, *p* < 0.05).

	PA		GC		RE		VA	
Thickness	Immediate	Aged	Immediate	Aged	Immediate	Aged	Immediate	Aged
0 mm	67.3(4.1) AB	66.8(2.9) a	72.5(5.6) A	72.7(6.2) a	67.4(3.6) A	69.1(3.3) ab	48.0(2.8) A	48.3(4.2) a
1.5 mm	68.3(3.3) A	65.0(2.9) a	71.4(4.5) A	71.2(3.8) ab	68.9(3.3) A	68.9(4.8) ab	49.1(2.6) A	48.4(5.4) a
3.5 mm	36.2(2.8) BC *	63.2(4.0) ab *	67.1(4.4) AB	66.4(2.8) ab	63.5(3.9) AB	71.8(3.3) a	48.3(3.2) A	49.5(4.4) a
5.5 mm	23.8(5.6) C *	58.6(2.6) abc *	51.5(4.6) BC	63.8(8.4) ab	62.0(4.9) AB *	70.5(1.8) a *	46.4(8.6) A	47.3(4.0) a
7.5 mm	U.C.	51.3(3.4) bc	20.7(6.0) C *	65.2(3.6) ab *	47.3(4.9) B	60.1(9.3) b	U.C.	U.C.
9.5 mm	U.C.	48.0(5.8) c	U.C.	62.8(8.4) b	U.C.	65.1(5.9) ab	U.C.	U.C.
Dark cure	U.C.	50.0(5.4) c	U.C.	62.6(4.8) b	U.C.	61.0(6.5) b	U.C.	U.C.

U.C.: All specimens in that condition remained uncured and were unmeasurable (*n* = 0 for DC; original group size, *n* = 7).

**Table 3:** Vickers hardness (VH) of dual-cure resin cements (PA, GC, RE) and light-cure resin cement (VA) polymerized by light irradiation through CAD-CAM resin composite plates of different thicknesses in the immediate and aged groups. The direct-light (0 mm) group represents specimens directly irradiated without an interposed CAD-CAM resin composite plate. The no-light (dark cure) group represents specimens polymerized without light irradiation. Values represent means ± standard deviations. Values followed by different uppercase and lowercase letters indicate statistically significant differences among plate thicknesses within the immediate and aged groups, respectively (Dunn’s post hoc test with Holm adjustment, *p* < 0.05). Groups sharing the same letter do not differ significantly. An asterisk (*) indicates statistically significant differences between the immediate and aged groups at the same plate thickness and for the same luting agent (Wilcoxon rank-sum test, *p* < 0.05).

	PA		GC		RE		VA	
Thickness	Immediate	Aged	Immediate	Aged	Immediate	Aged	Immediate	Aged
0 mm	31.8(2.1) A	34.8(2.2) a	32.1(2.7) A *	44.0(2.4) a *	17.7(3.0) A *	29.3(1.2) a *	22.6(1.9) A *	28.2(1.2) a *
1.5 mm	32.2(2.0) A	35.2(1.8) a	31.7(2.0) A *	41.9(2.7) abc *	18.1(2.5) A *	28.5(1.1) ab *	22.3(1.7) A *	27.8(2.1) a *
3.5 mm	24.0(1.6) B *	34.9(1.6) a *	31.1(3.2) A *	43.1(2.0) ab *	17.9(1.8) A *	29.3(1.5) a *	22.0(2.0) A *	28.7(1.8) a *
5.5 mm	N.D.	31.8(1.5) ab	18.0(1.4) B *	36.5(3.2) c *	N.D.	27.7(1.2) abc	23.1(2.1) A	23.0(1.5) b
7.5 mm	U.C.	30.8(1.8) ab	N.D.	36.8(2.2) c	N.D.	22.9(1.7) bc	U.C.	U.C.
9.5 mm	U.C.	28.8(1.3) b	U.C.	35.9(3.7) c	U.C.	22.4(1.2) bc	U.C.	U.C.
Dark cure	U.C.	29.0(1.0) b	U.C.	37.1(2.5) bc	U.C.	22.9(1.3) b	U.C.	U.C.

N.D.: Vickers hardness could not be determined because the indentation size exceeded the measurable range of the tester (<10 HV). U.C.: All specimens in that condition remained uncured and were unmeasurable (*n* = 0 for VH; original group size, *n* = 7).

## Data Availability

The original contributions presented in this study are included in the article. Further inquiries can be directed to the corresponding author.

## References

[1] Mannocci F., Bhuva B., Roig M., Zarow M., Bitter K., European Society of Endodontology (2021). European Society of Endodontology position statement: The restoration of root filled teeth. Int. Endod. J..

[2] Bindl A., Mormann W.H. (1999). Clinical evaluation of adhesively placed Cerec endo-crowns after 2 years--preliminary results. J. Adhes. Dent..

[3] Thomas R.M., Kelly A., Tagiyeva N., Kanagasingam S. (2020). Comparing endocrown restorations on permanent molars and premolars: A systematic review and meta-analysis. Br. Dent. J..

[4] Samra N., Madina M.M., El-Negoly S.A.E., Dawood L. (2024). The effect of restorative material selection and cementation procedures on the durability of endocrowns in the anterior teeth: An in-vitro study. BMC Oral Health.

[5] Elashmawy Y., Elshahawy W. (2023). Effect of Thermomechanical Fatigue Loading on the Internal and Marginal Adaptation of Endocrowns Utilizing Different CAD/CAM Restorative Materials. Int. J. Prosthodont..

[6] Pilecco R.O., da Rosa L.S., Baldi A., Machry R.V., Tribst J.P.M., Valandro L.F., Kleverlaan C.J., Scotti N., Pereira G.K.R. (2024). How do different intraoral scanners and milling machines affect the fit and fatigue behavior of lithium disilicate and resin composite endocrowns?. J. Mech. Behav. Biomed. Mater..

[7] AlDabeeb D.S., Alakeel N.S., Al Jfshar R.M., Alkhalid T.K. (2023). Endocrowns: Indications, Preparation Techniques, and Material Selection. Cureus.

[8] Jalali S., Asgari N., Pirooz P., Younespour S., Atri F. (2024). Comparison of Clinical Efficacy of CAD CAM Endocrowns Made of Feldspathic Ceramic, Zirconia Lithium Silicate, and Lithium Disilicate: A Two-Year Mixed Cohort Study. J. Dent..

[9] Kanat-Erturk B., Saridag S., Koseler E., Helvacioglu-Yigit D., Avcu E., Yildiran-Avcu Y. (2018). Fracture strengths of endocrown restorations fabricated with different preparation depths and CAD/CAM materials. Dent. Mater. J..

[10] Dartora N.R., Mauricio Moris I.C., Poole S.F., Bacchi A., Sousa-Neto M.D., Silva-Sousa Y.T., Gomes E.A. (2021). Mechanical behavior of endocrowns fabricated with different CAD-CAM ceramic systems. J. Prosthet. Dent..

[11] AboElhassan R.G., Watts D.C., Alamoush R.A., Elraggal A. (2024). Biomechanical behavior and Weibull survival of CAD-CAM endocrowns with different marginal designs: A 3D finite element analysis. Dent. Mater..

[12] Jin F., Yu X., Zhou H., Zhou J., Yang J., Luo Y., Chen Z. (2024). Fracture resistance of CAD/CAM endocrowns made from different materials in maxillary premolar interproximal defects. Clin. Oral Investig..

[13] El-Ma’aita A., Al-Rabab’ah M.A., Abu-Awwad M., Hattar S., Devlin H. (2021). Endocrowns Clinical Performance and Patient Satisfaction: A Randomized Clinical Trial of Three Monolithic Ceramic Restorations. J. Prosthodont..

[14] Keskin D.E., Saglam G., Geduk S.E. (2025). Effect of conventional and digital fabrication techniques on marginal and internal fit of lithium disilicate endocrowns. BMC Oral Health.

[15] AlHelal A.A. (2024). Biomechanical behavior of all-ceramic endocrowns fabricated using CAD/CAM: A systematic review. J. Prosthodont. Res..

[16] Jalalian E., Zarbakhsh A., Khorshidi S., Golalipour S., Mohammadnasl S., Sayyari M. (2024). Comparative analysis of endocrown fracture resistance and marginal adaptation: CAD/CAM technology using lithium disilicate vs. zirconia-reinforced lithium silicate ceramics. Saudi Dent. J..

[17] Saker S., Alqutaibi A.Y., Alghauli M.A., Hashem D., Borzangy S., Farghal A.E., Alnazzawi A.A., Ainoosah S., AbdElaziz M.H. (2024). The Influence of Ferrule Design and Pulpal Extensions on the Accuracy of Fit and the Fracture Resistance of Zirconia-Reinforced Lithium Silicate Endocrowns. Materials.

[18] Al Fodeh R.S., Al-Johi O.S., Alibrahim A.N., Al-Dwairi Z.N., Al-Haj Husain N., Ozcan M. (2023). Fracture strength of endocrown maxillary restorations using different preparation designs and materials. J. Mech. Behav. Biomed. Mater..

[19] Dong X., Ban J., Guo H., Zeng Z., Ren N., Bai S., Wang Z. (2025). Optimization of endocrown design parameters for mandibular second molars: A 3D finite element analysis. J. Mech. Behav. Biomed. Mater..

[20] Zhu J., Rong Q., Wang X., Gao X. (2017). Influence of remaining tooth structure and restorative material type on stress distribution in endodontically treated maxillary premolars: A finite element analysis. J. Prosthet. Dent..

[21] Tribst J.P.M., Dal Piva A.M.O., Madruga C.F.L., Valera M.C., Borges A.L.S., Bresciani E., de Melo R.M. (2018). Endocrown restorations: Influence of dental remnant and restorative material on stress distribution. Dent. Mater..

[22] Badawy H., Abo El-Farag S., Attia A. (2025). Two-Year Clinical Evaluation of Enamel Wear Antagonistic to Polished and Glazed Monolithic Zirconia Endocrowns. J. Esthet. Restor. Dent..

[23] Mahfouz Omer S.M., El-Desouky S.S., El-Saady Badawy R., Hadwa S.M., Ali Abdel Latif R.M. (2024). Qualitative surface roughness of lithium disilicate endo-crown for pulpotomized primary molars. Sci. Rep..

[24] Skorulska A., Piszko P., Rybak Z., Szymonowicz M., Dobrzynski M. (2021). Review on polymer, ceramic and composite materials for CAD/CAM indirect restorations in dentistry-application, mechanical characteristics and comparison. Materials.

[25] Hassan S.A., Beleidy M., El-Din Y.A. (2022). Biocompatibility and Surface Roughness of Different Sustainable Dental Composite Blocks: Comprehensive In Vitro Study. ACS Omega.

[26] Yuen J.J.X., Saw Z.K., Chua H.S., Beh Y.H. (2025). Clinical behavior and survival of CAD-CAM resin nanoceramic and polymer interpenetrating ceramic network material restorations on endodontically treated teeth: A systematic review and meta-analysis. J. Prosthet. Dent..

[27] Turker I., Kursoglu P. (2021). Wear evaluation of CAD-CAM dental ceramic materials by chewing simulation. J. Adv. Prosthodont..

[28] Amer D.M., Abdellatif A.M. (2024). In vivo evaluation of the enamel wear of primary molar against four types of crowns using the intra-oral scanner. BMC Oral Health.

[29] Jin C., Deng J., Pan P., Xiong Y., Zhu L., Gao S. (2023). Comparative study on the impact-sliding wear behaviour of CAD/CAM resin-ceramic materials and tooth enamel. Dent. Mater..

[30] Tribst J.P.M., Borges A.L.S., Silva-Concilio L.R., Bottino M.A., Ozcan M. (2021). Effect of Restorative Material on Mechanical Response of Provisional Endocrowns: A 3D-FEA Study. Materials.

[31] Zheng Z., Sun J., Jiang L., Wu Y., He J., Ruan W., Yan W. (2022). Influence of margin design and restorative material on the stress distribution of endocrowns: A 3D finite element analysis. BMC Oral Health.

[32] Zheng Z., He Y., Ruan W., Ling Z., Zheng C., Gai Y., Yan W. (2021). Biomechanical behavior of endocrown restorations with different CAD-CAM materials: A 3D finite element and in vitro analysis. J. Prosthet. Dent..

[33] Dikici B., Can E., Turkes Basaran E., Barut G., Donmez N. (2024). Fracture strength of endocrowns after thermomechanical aging. Odontology.

[34] de Kuijper M., Ong Y., Gerritsen T., Cune M.S., Gresnigt M.M.M. (2021). Influence of the ceramic translucency on the relative degree of conversion of a direct composite and dual-curing resin cement through lithium disilicate onlays and endocrowns. J. Mech. Behav. Biomed. Mater..

[35] David-Perez M., Ramirez-Suarez J.P., Latorre-Correa F., Agudelo-Suarez A.A. (2022). Degree of conversion of resin-cements (light-cured/dual-cured) under different thicknesses of vitreous ceramics: Systematic review. J. Prosthodont. Res..

[36] Gregor L., Bouillaguet S., Onisor I., Ardu S., Krejci I., Rocca G.T. (2014). Microhardness of light- and dual-polymerizable luting resins polymerized through 7.5-mm-thick endocrowns. J. Prosthet. Dent..

[37] Daher R., Ardu S., Kleverlaan C.J., DiBella E., Feilzer A.J., Krejci I. (2020). Effect of light-curing time on microhardness of a restorative bulk-fill resin composite to lute CAD-CAM resin composite endocrowns. Am. J. Dent..

[38] Ikemoto S., Komagata Y., Yoshii S., Masaki C., Hosokawa R., Ikeda H. (2024). Impact of CAD/CAM Material Thickness and Translucency on the Polymerization of Dual-Cure Resin Cement in Endocrowns. Polymers.

[39] Viljanen E.K., Skrifvars M., Vallittu P.K. (2004). Degree of conversion of a copolymer of an experimental monomer and methyl methacrylate for dental applications. J. Appl. Polym. Sci..

[40] Perea-Lowery L., Gibreel M., Garoushi S., Vallittu P., Lassila L. (2023). Evaluation of flexible three-dimensionally printed occlusal splint materials: An in vitro study. Dent. Mater..

[41] Vervack V., De Coster P., Vandeweghe S. (2021). Clinical Evaluation of Resin Composite CAD/CAM Restorations Placed by Undergraduate Students. J. Clin. Med..

[42] Vervack V., Keulemans F., Hommez G., De Bruyn H., Vandeweghe S. (2022). A completely digital workflow for nanoceramic endocrowns: A 5-year prospective study. Int. J. Prosthodont..

[43] Ibrahem S., Morad L., Husein H.A. (2024). Clinical Performance of Computer-Aided Design (CAD)/Computer-Aided Manufacturing (CAM) Resin Nanoceramic Endocrowns in Restoring Molars: An In Vivo Study. Cureus.

[44] Keskin S.C., Sakar A., Bolay S. (2024). A 3-year clinical evaluation of endocrown restorations with two different materials using the computer-aided design/ computer-aided manufacture system. J. Dent..

[45] Do T.T., Trinh T.M., Tran T.T.P., Nguyen V.T.T., Le L.N. (2024). Clinical performance of computer-aided design/computer-aided manufacture lithium disilicate ceramic endocrown restorations: A 2-year study. J. Conserv. Dent. Endod..

[46] Beji Vijayakumar J., Varadan P., Balaji L., Rajan M., Kalaiselvam R., Saeralaathan S., Ganesh A. (2021). Fracture resistance of resin based and lithium disilicate endocrowns. Which is better?—A systematic review of in-vitro studies. Biomater. Investig. Dent..

[47] Magne P., Carvalho A.O., Bruzi G., Anderson R.E., Maia H.P., Giannini M. (2014). Influence of no-ferrule and no-post buildup design on the fatigue resistance of endodontically treated molars restored with resin nanoceramic CAD/CAM crowns. Oper. Dent..

[48] Ghajghouj O., Tasar-Faruk S. (2019). Evaluation of Fracture Resistance and Microleakage of Endocrowns with Different Intracoronal Depths and Restorative Materials Luted with Various Resin Cements. Materials.

[49] Zheng Z., Wang H., Mo J., Ling Z., Zeng Y., Zhang Y., Wang J., Yan W. (2022). Effect of virtual cement space and restorative materials on the adaptation of CAD-CAM endocrowns. BMC Oral Health.

[50] Babaier R., Haider J., Silikas N., Watts D.C. (2022). Effect of CAD/CAM aesthetic material thickness and translucency on the polymerisation of light- and dual-cured resin cements. Dent. Mater..

[51] Dal Piva A.M.O., Verhoeff H., da Rosa L.S., Pereira G.K.R., Kleverlaan C.J., Tribst J.P.M. (2025). Optical properties of advanced lithium disilicate. Dent. Med. Probl..

[52] Kooshki F., Haeri Boroojeni H.S., Shekarchi F., Rahimi R. (2025). Fracture resistance in severely damaged primary maxillary central incisors restored with glass fiber and composite posts: An in vitro study. Dent. Med. Probl..

[53] Carek A., Dukaric K., Miler H., Marovic D., Tarle Z., Par M. (2022). Post-Cure Development of the Degree of Conversion and Mechanical Properties of Dual-Curing Resin Cements. Polymers.

[54] Aldhafyan M., Silikas N., Watts D.C. (2022). Influence of curing modes on conversion and shrinkage of dual-cure resin-cements. Dent. Mater..

[55] Benkeser S.M., Karlin S., Rohr N. (2024). Effect of curing mode of resin composite cements on water sorption, color stability, and biaxial flexural strength. Dent. Mater..

[56] Komagata Y., Nagamatsu Y., Ikeda H. (2023). Comparative Bonding Analysis of Computer-Aided Design/Computer-Aided Manufacturing Dental Resin Composites with Various Resin Cements. J. Compos. Sci..

[57] Yoshihara K., Nagaoka N., Benino Y., Nakamura A., Hara T., Maruo Y., Yoshida Y., Van Meerbeek B. (2021). Touch-Cure Polymerization at the Composite Cement-Dentin Interface. J. Dent. Res..

[58] Dimitriadi M., Petropoulou A., Zinelis S., Eliades G. (2024). Degree of conversion of dual-cured composite luting agents: The effect of transition metal-based touch-cure activators. J. Dent..

